# *Orientia tsutsugamushi*: A life between escapes

**DOI:** 10.1002/mbo3.1380

**Published:** 2023-09-10

**Authors:** Lea Fromm, Jonas Mehl, Christian Keller

**Affiliations:** ^1^ Institute of Virology Philipps University Marburg Marburg Germany

**Keywords:** budding, *Orientia tsutsugamushi*, phagosomal escape, scrub typhus

## Abstract

The life cycle of the mite‐borne, obligate intracellular pathogen *Orientia tsutsugamushi* (*Ot*), the causative agent of human scrub typhus, differs in many aspects from that of other members of the Rickettsiales order. Particularly, the nonlytic cellular exit of individual *Ot* bacteria at the plasma membrane closely resembles the budding of enveloped viruses but has only been rudimentarily studied at the molecular level. This brief article is focused on the current state of knowledge of escape events in the life cycle of *Ot* and highlights differences in strategies of other rickettsiae.

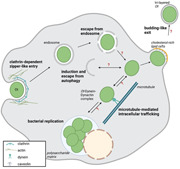

## 1 INTRODUCTION

To replicate within host cells, intracellular bacteria have evolved different strategies to invade, replicate, persist in, and eventually exit from their hosts. The intracellular lifestyle requires consecutive exit events, in which microorganisms must overcome host cell membranes, and enter and adapt to new compartments. These exit events are critical steps in the microbial life cycle and are usually required for bacterial replication and spread.

Current concepts on the exit events of intracellular bacteria have been derived from the thorough study of model organisms, such as *Salmonella*, *Shigella, Listeria*, or *Mycobacterium* species. In contrast, the exit strategies of many nonmodel—yet often medically relevant—bacterial species are so far only known in broad outline.

The causative agent of scrub typhus, *Orientia tsutsugamushi* (*Ot*), which belongs to the order of Rickettsiales, is an important but neglected human pathogen whose exit mechanisms have been poorly characterized. This is due to the lack of tools for the genetic manipulation of *Ot* and to the experimental challenges of working with a biosafety level 3 pathogen (Salje, 2017). A refined knowledge of this pathogen's exit strategies could define new targets for therapeutic approaches, for example, against long‐term persistence. This brief article summarizes the current state of knowledge, highlights commonalities and relevant differences to other intracellular bacteria, and defines questions and possible novel approaches to decipher the exit mechanisms of *Ot*.

## 2 *ORIENTIA TSUTSUGAMUSHI* AND SCRUB TYPHUS

*Ot* is a vector‐borne pathogen that is transmitted by the larval stage of trombiculid mites and causes potentially lethal febrile infection in humans (Luce‐Fedrow et al., 2018). About one million cases of scrub typhus are estimated annually by the World Health Organization, mainly in Southeast Asia (Bonell et al., 2017). In its vector mites, it is maintained as an endosymbiont by transovarian and transstadial transmission (Rapmund et al., 1969; Sonthayanon et al., 2010; Takhampunya et al., 2016). *Ot* was detected in the cytoplasm of salivary gland cells of infected mites by transmission electron microscopy (TEM). It has been shown that the feeding process of mites triggers the exit of *Ot* from these salivary gland cells, thus enabling access of bacteria to the acinar lumen and eventually transmission to humans and other potential hosts (Kadosaka & Kimura, 2003).

At the cutaneous site of transmission, where an eschar is formed in humans, *Ot* is mainly found in monocytes/macrophages and dendritic cells (Paris et al., 2012). From the dermal inoculation site, *Ot* disseminates systemically, as shown in animal models (Jiang et al., 2018; Keller et al., 2014; Soong et al., 2016), and may subsequently infect endothelial cells, cardiomyocytes, or hepatocytes (Moron et al., 2001; Pongponratn et al., 1998). This dissemination was shown to be mediated by CCR7‐expressing dendritic cells (Choi et al., 2013; Liang et al., 2022), rather than by CCR2‐expressing monocytes (Petermann et al., 2021). Latent persistence, remaining controlled by adaptive immunity (Hauptmann et al., 2016), is a common observation despite antimicrobial treatment (Chung et al., 2012; Kock et al., 2018).

**Figure 1:**
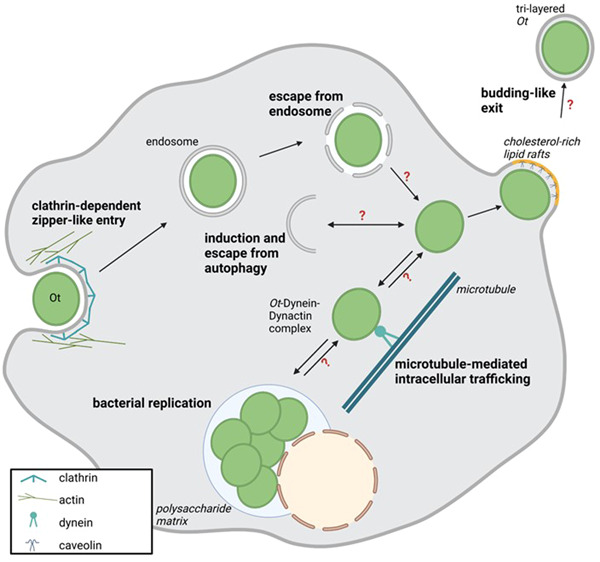
Intracellular life cycle of *Orientia tsutsugamushi* (*Ot*). Schematic overview of the intracellular replication cycle of *Ot*. Question marks indicate steps with hypothetical interactions and mechanisms.

*Ot* is a Gram‐negative bacterium with an obligate intracellular lifestyle (Figure 1). While its adaptation to the cytosolic environment resembles that of other rickettsiae, some biological aspects are fundamentally different from their close relatives: *Ot* lacks classical bacterial pathogen‐associated molecular patterns such as lipopolysaccharide or peptidoglycan (Amano et al., 1987; Atwal et al., 2017). *Ot*, in contrast to *Rickettsia* spp., does not cause early lytic cell death, leaving its host cell intact for more than 7 days of infection.

As *Ot* relies on intact cells for its replication, it has evolved different tools to maintain the host cell's integrity. One of these is its ability to inhibit apoptosis at the early stages of infection. This likely occurs through a delay in intracellular Ca^2+^ mobilization, possibly mediated by heat‐stable bacterial molecules (Kim et al., 2002). In turn, as the infection progresses, *Ot* induces a proapoptotic gene program that appears to outweigh its intrinsic antiapoptotic activity (Tantibhedhyangkul et al., 2011).

Another strategy to sustain the host cell's intactness is the nonlytic exit at the plasma membrane. Individual bacteria can leave the cell via a budding‐like mechanism to spread infection to other cells (Salje, 2021). Recently, the intra‐ and extracellular stages of *Ot* were shown to be fundamentally different with respect to morphology, transcriptional activity, and protein expression (Atwal et al., 2022).

Throughout its life cycle, *Ot* must repeatedly escape from membranous compartments for continued replication in different hosts and host cells: (1) from the phagosome to the cytoplasm, (2) in the cytosol from potential entrapment and degradation by autophagosomes, and (3) from the cytoplasm into the extracellular space via the plasma membrane.

## 3 THE PHAGOSOMAL ESCAPE OF *ORIENTIA TSUTSUGAMUSHI*

Attachment of *Ot* to its host cell involves interaction with heparan sulfate and syndecan‐4 as host cell receptors (Ihn et al., 2000; Kim et al., 2004), while invasion requires the interaction of the bacterial surface proteins TSA56 (type‐specific antigen of 56 kDa molecular weight) (Lee et al., 2008) and ScaC (Ha et al., 2011) with fibronectin. The entry follows a clathrin‐mediated zipper‐like and lipid raft‐independent mechanism (Kim et al., 2013). Within the host cell, *Ot* first localizes to clathrin‐ and adaptin‐positive compartments and recruits actin (Cho, Cho, Seong, et al., 2010; Chu et al., 2006). Subsequently, *Ot* is found in EEA1‐positive early endosomes, which mature into LAMP2‐positive endosomes. At about 2 h postinfection (p. i.), *Ot* escapes into the cytosol and traffics to the perinuclear region where it initiates replication (Kim, Ihn, Han, Seong, Kim, Choi, 2001).

In *Ot*, the phagosomal escape has not been mechanistically elucidated. However, recent proteomic and functional studies have provided possible clues derived from the related *Rickettsia* spp. These bacteria disrupt phagosomal membranes with the help of different membranolytic effector proteins, for example, the hemolysin TlyC or the phospholipase D (Pld) (Housley et al., 2011; Radulovic et al., 1999; Rahman et al., 2013). Genomic studies among 55 *Rickettsia* strains revealed high conservation of *tlyC* and *pld* genes (Gillespie et al., 2015). Expression of TlyC from *Rickettsia typhi* in an otherwise nonhaemolytic *Proteus* species conferred a membranolytic phenotype (Radulovic et al., 1999). Furthermore, the ability of Pld to reach the periplasm was interpreted to be consistent with outer membrane localization or secretion, thus enabling membranolytic activity (Ammerman et al.,©^2008^; Gillespie et al., 2015). Concordantly, ectopic expression of the *pld* and *tlyC* genes of *R. prowazekii* in *Salmonella enterica*, which is usually trapped in phagosomes, mediated the phagosomal escape of the transformed *Salmonella* bacteria (Whitworth et al., 2005). In addition, *R. prowazekii* and *R. conorii* treated with an antibody directed against Pld showed a reduced cytotoxic effect on Vero cells (Renesto et al., 2003), further highlighting its role as a likely virulence factor in *Rickettsia* infection.

**Figure 2:**
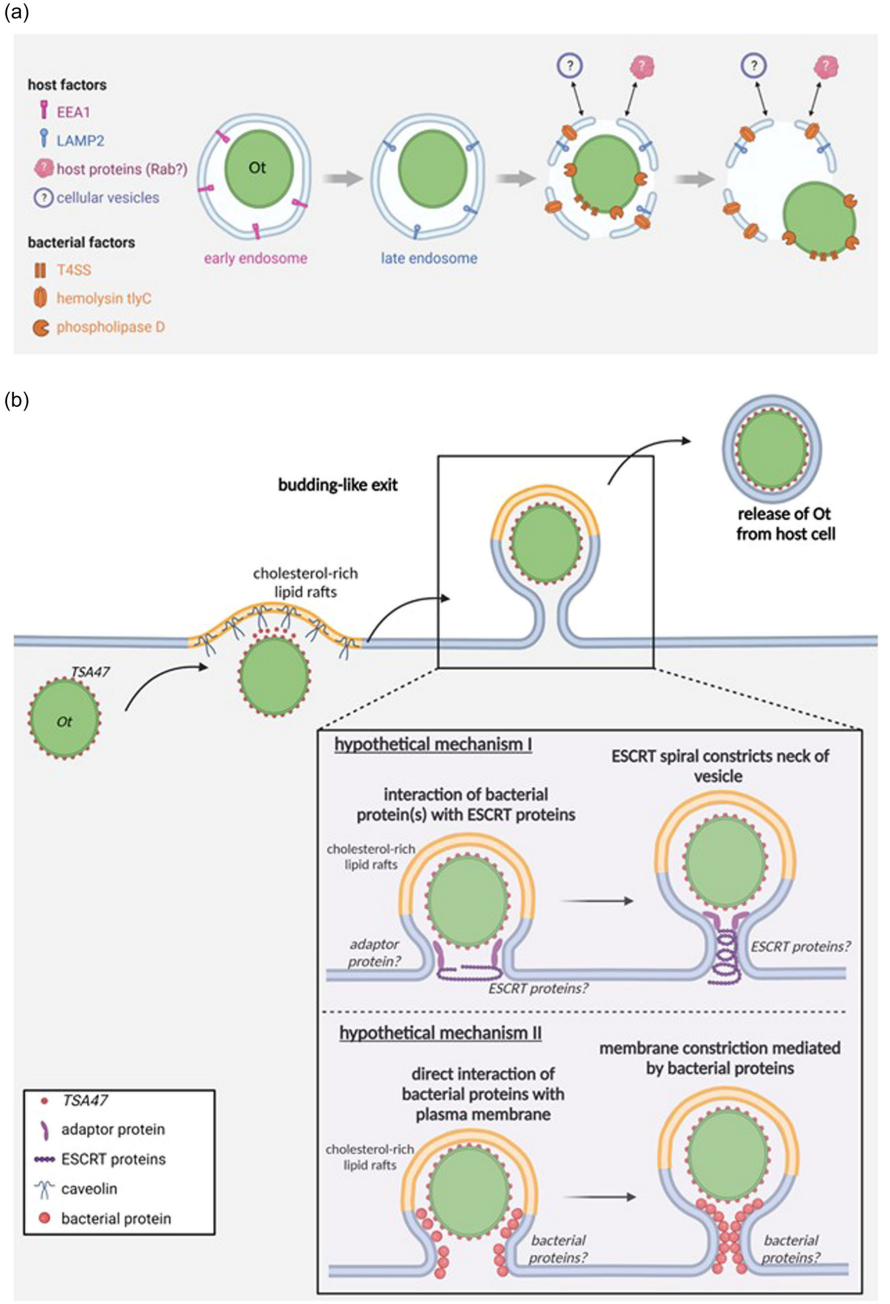
Endosomal escape and budding‐like cellular exit of *Orientia tsutsugamushi* (*Ot*). (a) *Ot* escapes from late endosomes. T4SS, type IV secretion system. Question marks indicate steps with hypothetical interactions and mechanisms. (b) Cholesterol‐rich lipid rafts (yellow) and the bacterial protein TSA47 (red) are involved in the budding‐like exit of *Ot*. The lipid and protein composition of the acquired membrane layer are so far unknown. The large inset depicts two hypothetical mechanisms of the budding process, involving the cellular ESCRT machinery (I) or facilitation by bacterial outer membrane proteins (II). EEA1, early endosome antigen 1; ESCRT, endosomal sorting complex required for transport; LAMP2, lysosome‐associated membrane protein 2; TSA47, type‐specific antigen 47.

Indeed, *tlyC* and *pld* were also identified in the genome of *Ot* (Cho et al., 2007), and both proteins were found expressed in proteomic analyses of *Ot*‐infected cells (Atwal et al., 2022; Cho, Cho, Min, et al., 2010). In the absence of direct experimental evidence, it was thus speculated that, in analogy to other *Rickettsia* spp., these proteins might be involved in the disruption of the phagosomal membrane in *Ot* infection (Figure 2a) (Ge & Rikihisa, 2011).

Moreover, in inhibition experiments with bafilomycin A, an inhibitor of the vacuolar H^+^‐ATPase that blocks lysosome acidification (Bowman et al., 1988), it was observed that a low endosomal pH is required for the escape of *Ot* to the cytosol and subsequent cytosolic replication (Chu et al., 2006). While mechanistic details on the contribution of host factors to the escape of *Ot* are still lacking, it is known from infection of epithelial cells by *Shigella flexneri* that the invasion does not only involve the formation of a bacteria‐containing vacuole but also triggers the recruitment of further cellular factors, for example, macropinosomes and small Rab GTPases (Weiner et al., 2016). Such host factors could additionally contribute to the phagosomal escape of *Ot* (Figure 2a). Transgenic approaches in recently developed reporter systems for cytosolic access or lysosomal damage, such as galectin‐3 reporter cells (Chang et al., 2020) or cells transgenic for a fluorescent sphingomyelin‐binding equinatoxin II (Niekamp et al., 2022), are likely to facilitate mechanistic studies.

## 4 ESCAPE FROM CELLULAR AUTOPHAGY

Once released to the cytosol, *Ot* faces the next challenge on the way to successful replication: autophagy. In autophagy, cytosolic material is delivered to lysosomes for degradation, including soluble materials and proteins, organelles, and bacteria that have gained access to the cytosol. A number of intracellular bacteria have evolved mechanisms to evade autophagy, such as by inhibiting autophagosomes or evading recognition through camouflage. Interestingly, *Ot* was found to induce, but also actively evade, cellular autophagy to avoid bacterial elimination (Ko et al., 2013). While the induction of autophagy by *Ot* was found independent of bacterial viability, only living bacteria can successfully escape autophagosomal degradation (Choi et al., 2013). Blocking of bacterial translation with antibiotics leads to the trapping of bacteria in autophagosomes and their degradation in the lysosome (Ko et al., 2013). This suggests that a bacterial protein likely mediates the active escape. Such candidates can be localized in the bacterial membranes and recruit host cell proteins to disguise bacteria from autophagic recognition, as, for example, described for the *Listeria* protein ActA (Yoshikawa et al., 2009). Alternatively, effector proteins may be secreted and counteract autophagy, as known from *Shigella* IscB, which competitively inhibits the autophagy‐inducer VirG (Ogawa et al., 2005). In *R. typhi*, the secreted protein Risk1 was recently shown to delay autophagic maturation by a phosphatidylinositol 3‐kinase‐dependent mechanism (Voss & Rahman, 2021).

Risk1 is a substrate for type IV secretion systems (T4SS), which are abundantly encoded in the genomes of both *Rickettsia* spp. and *Ot* (Cho et al., 2007; Gillespie et al., 2010). In fact, as one example, a *risk1* homolog is conserved in *Ot* and could have a similar role in protecting *Ot* from autophagy. The massive proliferation of conjugative systems in the *Ot* genome suggests the presence of a much higher number of so far unidentified T4SS effectors. The use of mass spectrometry analysis of immunoprecipitated T4SS could help to comprehensively identify new candidates. Blocking effectors in *Ot* infection with specifically raised antibodies will be instrumental in characterizing their function.

## 5 EXIT FROM THE CYTOPLASM

For intracellular motility, *Ot* and other Rickettsiales need to exploit the cellular cytoskeleton. In contrast to many other intracellular bacteria that induce actin tails, *Ot* utilizes microtubule‐mediated processes to propel itself to the replication site in the vicinity of the microtubule‐organizing center (Kim et al., 2001). After several days of replication, hitherto unknown mechanisms trigger the trafficking of *Ot* toward the plasma membrane.

Here, *Ot* initiates an unusual, nonlytic budding mechanism to exit its host cells, during which the exiting bacterium becomes encased by the host plasma membrane (Figure 2b) (Ewing et al., 1978; Tsuruhara et al., 1982; Urakami et al., 1984). In that regard, the exit of *Ot* is strikingly different from other Rickettsiales such as *R. rickettsii* or *R. typhi*, which spread from cell to cell by actin‐dependent protrusion followed by direct engulfment by neighboring cells (Van Hauptmann et al., 2017; Heinzen et al., 1993; Kirk et al., 2000), but also from the various exit strategies used by many other intracellular bacteria (Flieger et al., 2018).

Once at the plasma membrane, *Ot* bacteria assemble in clusters and within a very short distance of <30 nm from the membrane, as demonstrated by TEM (Urakami et al., 1984). These membrane‐associated bacteria slightly bulge the plasma membrane toward the extracellular space (Figure 2b). Depending on experimental conditions, budding is observed in vitro after 48–72 h postinfection (Tsuruhara et al., 1982; Urakami et al., 1984), and was also found in murine peritoneal mesothelial cells in vivo (Ewing et al., 1978). Most budding organisms have been observed in perpendicular orientation at the plasma membrane, although a minority of bacteria bud in horizontal direction (Tsuruhara et al., 1982). Interestingly, the ratio of membrane‐associated bacteria to the entire number of bacteria remained largely constant at 30%–35% (Urakami et al., 1984).

Of note, budding of host membrane‐enveloped *Ot* was also observed in vector mites, in particular in the salivary glands of adults, from where *Ot* is released during vector feeding (Kadosaka & Kimura, 2003), and in the rudimentary reproductive organs of larvae (Tamura et al.,©^1994^; Urakami et al.,©^1994^). Thus, the cellular exit of *Ot* via budding seems not restricted to mammalian infections but is likely a conserved hallmark of susceptible vertebrate and nonvertebrate eukaryotic hosts.

Ultrastructural studies have demonstrated the formation of a tight budding neck that forms between the exiting bacterium and the plasma membrane and contains a cytoplasmic core (Figure 2b) (Ewing et al., 1978). However, it has not been elucidated which bacterial or cellular factors control its formation.

During the cellular exit process, *Ot* associates with cholesterol‐rich lipid rafts and co‐localizes with caveolin at the plasma membrane (Figure 2b). The bacterial TSA47 surface protein was hypothesized to be involved in this interaction (Kim et al., 2013). TSA47 is a homolog of the human serine protease htrA1 and an integral *Ot* membrane protein cross‐linked to other bacterial surface proteins, for example, TSA56, and was observed to be in part secreted during that exit process.

While budding, *Ot* becomes covered by a third membrane layer that is derived from the host cell's plasma membrane. Since trilayered *Ot* are visible outside the host cell after the exit process (Kadosaka & Kimura, 2003) and can even be found in the phagosomes of newly infected cells (Ewing et al., 1978; Rikihisa & Ito, 1980), it is assumed that the additional membrane is not quickly removed upon exit. From observations in mouse fibroblasts, two different modes of removal have been suggested: The encasing third membrane layer can be lost either outside the host cell or is removed in the process of *Ot* gaining access to the host cell's cytoplasm, for example, in the phagosome or during phagosomal escape (Ewing et al., 1978; Kadosaka & Kimura, 2003; Urakami et al., 1984). The process details remain to be elucidated.

## 6 MECHANISMS OF MEMBRANE SCISSION: IMPLICATIONS FOR THE BUDDING OF *ORIENTIA TSUTSUGAMUSHI*

The exit process of *Ot* is highly reminiscent of the budding mechanism of many enveloped viruses, where nascent virions acquire lipid bilayers from the host and induce membrane scission upon the formation of a small budding neck at the plasma membrane. The molecular mechanisms of viral exits have been extensively studied in the past 20 years and could provide clues on how *Ot* buds from the host cell.

In terms of localization, the budding of many enveloped viruses typically occurs from cholesterol‐rich, “lipid raft‐like” microdomains in the plasma membrane. It is assumed that these are generated by recruitment and coalescence from smaller pre‐existing rafts, or by de novo assembly (Lorizate & Krausslich, 2011).

Regarding temporal organization, the budding of enveloped viruses involves three sequential steps: (1) transport and assembly of the essential components to the budding site, (2) induction of membrane curvature by the nascent virion, and (3) scission of the virion away from the cellular membrane (Lorizate & Krausslich, 2011). The topology of viral budding has been termed “reverse”: In classical vesiculation events with “normal” topology (e.g., endocytosis), membrane constriction occurs *toward* the cytoplasm and is driven by proteins acting from the *outside* of the bud neck. In contrast, viral budding requires membrane scission *away* from the cytoplasm and the action of cytoplasmic factors from *within* the bud neck (Schöneberg et al., 2016; Votteler & Sundquist, 2013). Sharing the same membrane orientation as enveloped viruses, the budding of *Ot* thus also occurs in reverse topology.

Molecularly, “normal” and “reverse” topology scission differ fundamentally. While normal topology scission mainly involves cytosolic membrane coating by clathrin and membrane scission by the GTPase dynamin (Antonny et al., 2016), reverse topology scission is mainly driven by the ESCRT (endosomal sorting complexes required for transport) machinery (Schöneberg et al., 2016). The recruitment of ESCRT proteins (e.g., TSG101, Alix, or the NEDD4 family of ubiquitin ligases) occurs via “late domains” in viral structural proteins that mediate budding, such as the PT/SAP, PPXY, or YXXL motifs and others (Welker et al., 2021). First characterized in the budding of the human immunodeficiency virus (HIV‐1), it is now clear that the cellular ESCRT machinery is hijacked by a large number of enveloped viruses from different families for host cell exit (Votteler & Sundquist, 2013).

The critical property of the ESCRT machinery is to constrict membranes over a relatively large distance of sometimes 1.5 µm (Votteler & Sundquist, 2013). In the first step, early‐acting factors initiate ESCRT assembly through the interaction of cellular adaptor proteins with, for example, viral structural proteins. One well‐characterized adapter is Alix (first described as Bro1 in yeast), now known as a member of the Bro1 family of adapter proteins (Martin‐Serrano et al., 2003; McCullough et al., 2008). Such adapter proteins then allow the concentration of ESCRT‐I/‐II proteins (e.g., TSG101) in supercomplexes in the interior of the nascent budding neck. Together, these molecules act to recruit late‐acting factors, the ESCRT‐III proteins, which are mostly members of the CHMP family (Van Engelenburg et al., 2014; Meng et al., 2020). These proteins can form homo‐ and hetero‐oligomeric filaments that can further assemble into spiral helices (Lorizate & Krausslich, 2011). For actual membrane severing by the ESCRT machinery, it is thought that a dynamic change of ESCRT‐III composition in the budding neck, mediated by ATPase Vps4, drives membrane deformation and eventually scission (Lata et al., 2008; Maity et al., 2019; Pavlin & Hurley, 2020; Schöneberg et al., 2016).

The cellular exit of *Ot* occurs in reverse topology, with striking structural similarities to the budding of enveloped viruses, as, for example, described for Lassa virus or HIV‐1 (Figure 2b): First, bacteria located in close proximity to the plasma membrane induce a local membrane curvature (Tsuruhara et al., 1982; Urakami et al., 1984). Second, the budding bacteria get fully covered by a tight plasma membrane envelope, and a narrow budding neck in the plasma membrane is formed behind them (Ewing et al., 1978). Eventually, membrane‐encased bacteria are released into the extracellular space (Kadosaka & Kimura, 2003; Urakami et al., 1984). Another similarity lies in the association of bacterial proteins with lipid rafts late in infection: It was shown that *Ot*, and its two surface proteins TSA47 and TSA56, associate with caveolin‐rich membrane fractions after 7 days p. i., and this association was susceptible to treatment with the lipid raft‐disrupting agent methyl‐β‐cyclodextrin (Kim et al., 2013).

Based on these conspicuous similarities to viral budding, the ESCRT proteins known to mediate the budding of many enveloped viruses will be interesting candidates in studying the budding process of *Ot* (Figure 2b, hypothetical mechanism I). One aspect supporting a potential involvement of ESCRT is the presence of one or more YXXL motifs in the surface proteins TSA47, TSA56, or ScaC, which could predispose for binding to the adaptor protein Alix and subsequent budding (Strack et al., 2003). The use of lipid raft markers in super‐resolution or correlative live‐electron microscopic imaging as well as the state‐of‐the‐art study of interaction partners of *Ot* surface proteins will pave the way for this approach.

Reverse topology budding of enveloped viruses is, however, not exclusively dependent on the ESCRT machinery. In influenza virus infection, the viral M2 protein was shown to be sufficient to drive membrane scission for virion release (Rossman et al., 2010). *Ot* surface proteins, as membrane‐resident or shed proteins, or secreted effectors, could therefore also contribute to the budding process (Figure 2b, hypothetical mechanism II).

While no other intracellular bacteria are known to utilize budding for host cell exit, the knowledge of cellular or bacterial proteins in regulating intracellular bacterial infections is scarce. Some studies indicate a role for ESCRT in the earlier stages of infection, for example, in the sealing of membrane damages in the phagocytic vacuole (Göser et al., 2020; López‐Jiménez et al., 2018; Radulovic et al., 2018). Thus, the factors mediating the budding of *Ot* may be involved in more than one step of the infection cycle, or in other critical cellular processes such as cell division, which poses methodological challenges for experimental investigations. Inducible, tetracycline‐independent systems in which the expression of nonfunctional variants of candidate proteins can be switched on by the addition of specific ligands will help elucidate the role of cellular components during the third exit event of *Ot* (Chung et al., 2015; Kolesnikova et al., 2009).

In summary, crucial steps and mechanistic details in the exit mechanism of *Ot* remain to be elucidated. Future studies will need to address the question of which host cell factors are involved, and how far the exit is conserved in vertebrate and the invertebrate host.

## AUTHOR CONTRIBUTIONS

**Lea Fromm**: Visualization (lead); writing—original draft (equal); writing—review and editing (supporting). **Jonas Mehl**: Supervision (supporting); visualization (equal); writing—review and editing (supporting). **Christian Keller**: Conceptualization (lead); funding acquisition (lead); project administration (lead); supervision (lead); visualization (supporting); writing—original draft (equal); writing—review and editing (lead).

## CONFLICT OF INTEREST STATEMENT

None declared.

## ETHICS STATEMENT

None required.

## Data Availability

Not applicable.
